# Developing an appreciation of patient safety: analysis of interprofessional student experiences with health mentors

**DOI:** 10.1007/s40037-016-0258-4

**Published:** 2016-03-14

**Authors:** Sylvia Langlois

**Affiliations:** Centre for Interprofessional Education, University of Toronto, Toronto Western Hospital, Toronto, ON Canada

**Keywords:** Patient safety, Patient lived experiences, Health profession education

## Abstract

**Introduction:**

A critical task for health profession educators is to foster student appreciation of patient quality and safety issues. Although instructional methods vary, few focus on the direct communication of the patient experience to students. This qualitative study explores the experiences and learning of health profession students participating in a Safety Module in the Health Mentor Programme.

**Methods:**

Small interprofessional groups of students were paired with a health mentor, an individual experiencing chronic health challenges. Students followed a 90-minute, semi-structured interview format exploring issues regarding quality care and safety. Following the interviews, students participated in a facilitated asynchronous online discussion and completed a reflective practice paper. An inductive thematic analysis of both of these text-based datasets revealed emerging themes.

**Results:**

Themes identified in the data included: Patient partnerships as critical to optimal care; consideration of a variety of safety issues; importance of advocacy in promoting safety; improvement of future practice enabled through patient perspectives on clinical error; and embracing of interprofessional communication and collaboration.

**Conclusions:**

The findings suggest that engagement with the health mentor narratives facilitated students’ appreciation of quality and safety issues related to patient care.

## Essentials

- Students reported that partnerships between healthcare practitioners and patients are critical to the provision of optimal care
- Students recognized that safety issues go beyond the standard measures of medication errors, falls, infection rates and adverse incidents and include nuances such as stigma
- Students described the importance of advocating for patients and enabling empowerment so they could advocate for themselves to promote safe quality care
- Future practice was enabled through student reflection on patients’ perspectives of clinical error
- Students embraced interprofessional communication and collaboration as a strategy to improve quality and safety

## Introduction

A critical task for health profession educators is to foster student appreciation of patient quality and safety issues. Although instructional methods vary, few address the direct communication of the patient experience to students. This paper focuses on the learning and experiences of health profession students participating in a Safety Module as part of a Health Mentor Programme. In this context, health mentors are individuals living with chronic health challenges who share their experiences in the healthcare system with health profession students in order to contribute to their professional learning.

## Background

In 2000, the ground-breaking report, *To Err is Human*, highlighted the extent of error in the healthcare system, suggesting that between 44,000 and 98,000 people in American hospitals die from preventable mistakes [1]. Furthermore, lost income and higher health expenditures from longer lengths of stay, as well as higher infection and injury rates, are estimated to be between $ 17 and $ 29 billion USD [1, 2]. The pressing concern regarding safety in care is indeed a global problem, with reports of 10 % of patients impacted by errors worldwide [3]. Even with an enhanced focus on the creation of a culture of safety in healthcare organizations and efforts to promote teamwork to address quality and safety, much work is still needed [4].

With the growing awareness of unintended harm to patients in health delivery, educational institutions have been charged with exploring enhanced instructional approaches to prepare students to practice in a manner promoting safety [5]. The Institute for Healthcare Improvement has been instrumental in providing training in quality and safety for both students and professionals [6]. Moreover, the World Health Organization has published curriculum guides for both medical schools and other health profession programmes to facilitate integration of patient safety teaching into health profession curricula [7, 8]. Typically, patient safety education addresses topics such as healthcare systems, informatics, communication and teamwork skills, human factors science, management of errors, and medication safety. Team collaboration is recognized as a key method to improve patient safety; however, instruction continues to be primarily discipline specific [8–10]. Educational methods described in the literature include didactic lectures, intensive workshops, simulations with standardized patients and/or role plays, root cause analyses, and quality assurance projects, yet these pedagogical approaches are typically distant from clinical work and patient contact [10–12].

Authentic clinical experiences, viewed as critical to learning, are enhanced through student exposure to lived experiences and narrative [13]. Narratives have the potential to have a significant impact on practitioners, enabling the development of characteristics that are desirable—empathy, professionalism, trustworthiness and reflection [13]. Cognitive, affective and symbolic applications fostered by authentic experiences serve to promote transformation [13]. Thus, encounters with patients, whether in person or through written accounts, provide unique insights that foster learning.

Reflective capacity is an important skill for health practitioners; it is foundational to critical thinking and the clinical reasoning process, and contributes to the development of professionalism [14]. To foster reflective capacity a supportive environment, realistic context, group discussion and mentorship are required [14]. The link between reflection and patient safety has been explored; critical reflection seemed to have an impact on attitudes to patient safety as well as future intentions, whereas reflection was associated with an awareness of actions to take to promote patient safety [15]. This finding is substantiated through the work of Mezirow who discussed the relationship between critical reflection and transformative learning. Engaging in critical reflection permits the learner to analyze experiences and create new understandings that influence future behaviours and decision-making [16].

Although the literature identifies a variety of approaches, the experiences of health system users have not been described in relationship to the development of competencies pertaining to patient safety. This study explores the learning experiences of health profession students participating in a safety module as part of the Health Mentor Programme where learners engage with the stories of patients, discuss their perceptions in facilitated interprofessional groups and respond through reflective writing.

## Methods

### Design

This is a report of a qualitative study investigating the emerging themes from the online discussion text and students’ written reflective submissions. Research was conducted within a realist paradigm, based on the belief that there is an external reality of student experience. An inductive approach focusing on description was selected for analysis, where identified themes were linked to the dataset rather than to theoretical perspectives.

### Programme description

Students from the health profession programmes at the University of Toronto, Canada, may choose to participate in the Health Mentor Programme (one of many elective offerings) as part of the requisite Interprofessional Education curriculum that is incorporated into their uniprofessional programmes. The Health Mentor Programme, adapted from a similar programme at Thomas Jefferson University [17], is an elective learning activity underpinned by considering both transformative learning theory (students may experience transformative change as a result of their experiences) and social constructivism (students create meaning through interactions with the health mentor and peers). This programme pairs a group of students representing different professions with an individual referred to as a health mentor. Health mentors live in the community and have a variety of chronic health challenges resulting in multiple contacts with the healthcare system (Table 1). They share their experiences with health profession students to shape their understanding of the impact of chronic health challenges and contribute to the development of professional practice attitudes and behaviours. Faculty facilitators representing Medicine, Nursing, Occupational Therapy, Pharmacy, Physical Therapy and Speech-Language Pathology guided students through the asynchronous online discussions, helping them process what they had learned.

**Table 1:** Health challenges experienced by health mentors

Health challenge categories	Number of health mentors
Neurological conditions (including stroke, multiple sclerosis, amyotrophic lateral sclerosis, post polio, cerebral palsy)	9 adults and one parent caregiver
Multiple comorbidities (Including cardiac, gastrointestinal, cancer, reduced vision, diabetes)	5
Rheumatoid arthritis	3

The Health Mentor Programme consisted of four modules: (1) Chronic Health Challenge in Context; (2) Impact of the Chronic Health Challenge; (3) Ethical and Professionalism Issues; and (4) Patient Safety Issues. Over the course of 5 months, students met with their assigned health mentor for approximately 90 min on each of four occasions to conduct an interview using a semi-structured interview guide prepared by the programme organizer. Questions were developed through consultations with local experts. Topics for the Patient Safety Module included experience with hospitalization, transitions in care, falls, community living, home and workplace safety, medication use, impact of pain and alternative healthcare. Following each interview with their assigned health mentor, students participated in a facilitated online discussion to synthesize learning and consider issues and contributions interprofessionally.

The University of Toronto Ethics Board granted approval for this investigation. The work was carried out in accordance with the Declaration of Helsinki.

### Participants

Students participating in the Health Mentor Programme were recruited to take part in the study analyzing their experiences. The principal investigator provided information letters in advance. Students attended an orientation session, which included a review of the study requirements, distribution and collection of consent letters; all students registered in the Health Mentor Programme opted to participate in the study. Although the study included an analysis of the Ethics and Professionalism Module as well (reported elsewhere), this paper will only address investigation of the Patient Safety Module.

Originally, 118 first-year students from seven health professions registered. Over the course of 5 months, 27 students dropped out of the programme citing challenges with scheduling and course workload. No participating student specifically dropped out of the study. The data from the remaining 91 students (Table 2) who completed the Patient Safety Module were used for analysis.

**Table 2:** Student characteristics

Professional programme	Number of first-year students
Medicine	17 (13 female, 4 male)
Nursing	8 (all female)
Occupational therapy	16 (all female)
Pharmacy	18 (all female)
Physical therapy	14 (all female)
Social work	7 (6 female, 1 male)
Speech-language pathology	11 (10 female, 1 male)
**Total**	91

### Data collection

Student groups completed the interview with their health mentor using the interview guide provided. Following the encounter with the health mentor, students participated in the facilitated online discussions and submitted their one-page reflections on Blackboard, the university learning management system. Students were required to complete a minimum of three posts in response to the facilitator’s prompting questions (provided by the researcher) and fellow participants’ posts. Upon completion of the programme, the research assistant retrieved the datasets from Blackboard and ensured that all identifying information was removed for subsequent analysis.

### Qualitative analysis

Braun and Clarke [18] informed this inductive thematic analysis process, since this study focused on description of student experiences and their learning through the Health Mentor Programme rather than interpretation or construction of theory. The research assistant engaged in close reading of the dataset and identified initial similar units, which were then grouped into themes. Subsequently, the principal investigator reviewed the data, revised and verified initial codes. Both discussed and reconciled any differences. Ideas that reoccurred and pertained to the research questions were considered as emerging themes. Frequent consultations led to agreement of themes. They re-examined the dataset to determine if any other ideas were emerging and explored both external heterogeneity and internal homogeneity.

Researcher reflexivity was addressed throughout by recording impressions and values that might influence the process in a journal format [19]. The principal investigator and research assistant considered the assumption that student engagement with health mentors would influence their perceptions of patient safety and the way in which they might interpret the data during the analysis. Ongoing discussion with the research assistant provided the opportunity to reflect more deeply and to ensure that influence was minimized.

## Results

Five key themes emerged from the data. They included (1) Patient partnerships as critical to optimal care; (2) Consideration of a variety of safety issues; (3) Importance of advocacy in promoting safety; (4) Improvement of future practice enabled through patients’ perspectives on clinical error; (5) Embracing of interprofessional communication.

### Theme 1: Patient partnerships as critical to optimal care

Students identified that patient and family partnership is critical to the provision of optimal care in a complex health system and that healthcare professionals should work to establish partnerships with patients. One student noted:

For my own future practice, incidents such as these emphasize to me the need for healthcare professionals to focus on patient-centred care and to understand that the relationship is a partnership between the patient and the professional, and not one telling the other what to do and the other doing it without question.

They noted that health practitioners should listen to patients to understand their perspective. For example:

Some of the healthcare professionals whom she worked with failed to listen to her concerns and did not receive her feedback in a professional manner. For example, one member of her healthcare team at the time acted in a paternalistic and unprofessional fashion by stating that he “knew better than her” because he had had many years of education at university and extensive training, “which she did not have”.

### Theme 2: Consideration of a variety of safety issues

Students encountered a variety of safety issues in the narratives of the health mentors, and consequently broadened their understanding of safety to include behaviours and attitudes. For example:

I think it is important to look beyond the physical aspect of a “safe environment” and consider behaviours and attitudes that could impact the client…other individuals could be facing discrimination in their communities which could negatively impact their environment, making it more hostile than safe.

Many of the health mentors had been hospitalized and experienced challenges regarding safety when returning to their home environments. Students commented on the need for coordinated care for a safe discharge planning. One student noted:

It does not seem safe or fair to send a patient home who cannot cope and does not know who to contact for help. This is something the team should discuss and have at least one healthcare professional in charge of discussing this with the patient before the patient is discharged.

### Theme 3: Importance of advocacy in promoting safety

Reflecting on the health mentor experiences in the healthcare system helped students commit to advocating for patients to ensure safety. For example a student commented that:

I will speak up and advocate for my clients and let other healthcare professionals know that this is a very important concept…

Additionally, students recognized that they played a role in empowering patients to advocate for themselves, as they are the constant factor in the multiple interactions with healthcare providers. One student wrote:

I think that bridging the gap between professions may start with encouraging the patient to provide us with information, and advocate for themselves in the healthcare setting. This may be especially helpful in the community setting where we may not always have access to one another.

Students also noted that empowering patients to advocate for themselves may help them to identify as a member of the healthcare team. For example:

… we can help patients advocate for themselves by educating them that each professional they meet throughout their care is working as a team, and that as a patient, they are also part of that team. If this is explained to patients when they first start receiving care, and if it is reinforced by various members of the team, it seems likely that patients will gradually become more comfortable speaking up about their healthcare needs.

### Theme 4: Improvement of future practice enabled through patient perspectives on clinical error

Students heard a number of examples of clinical error and unprofessional behaviour in the care of the health mentors they interviewed. In considering these instances in their small group discussions and reflections, students commented on how these experiences would affect their own future behaviours related to safety. For example:

One incidence involved a nurse trying to administer medication secretly to the patient against the competent patient’s deliberate refusal. Another incidence involved a physiotherapist belittling and blaming the patient…I found both instances rather shocking, and would have wished that in those instances (if I were in the same position), the nurse/physiotherapist would have tried to understand the patient’s fears and respected the patient’s ability to read their own body before assuming they knew what was best and forcing it upon the patient/blaming the patient.

Safety and quality were also expressed in students’ comments regarding the need for health practitioners to ensure they are using current interventions, as demonstrated in this quote:

…it was realized that the medication did not do what it was supposed to and could have some complications for the patient’s diagnosis. This, in my mind, is unacceptable, and every healthcare professional should strive to stay current with e.g. medications, interventions, etc. and be able to share and discuss the key points with clients.

### Theme 5: Embracing interprofessional communication and collaboration

Communication and collaboration among healthcare team members was a frequent topic in discussion boards. Many students commented on the negative impact on safe and quality care as a result of miscommunication. For example:

I couldn’t believe that such a health risk from medications can come from miscommunication of healthcare professionals. This really drives the point home for me on the importance of interprofessional communication and teamwork: a client/patient’s psychological and physical well-being, as well as their life, depends on it.

On many occasions, students described the importance of healthcare team members working collaboratively and communicating well. For example:

I think that this emphasizes the importance of interprofessional care and communication between different professionals at the same and at different facilities.

## Discussion

Key themes emerging from the analysis of learners’ written reflections and online discussions were partnerships between patient and practitioner, appreciation of the breadth of safety issues, patient advocacy, reflections on future practice, and the need for interprofessional communication and collaboration.

Students discussed the impact of patient-provider partnerships or lack thereof in the context of their health mentor’s encounters with the healthcare system. They identified key concepts of dignity and respect, information sharing, patient and family participation in care and collaboration, also articulated in the Quality and Patient Safety Governance Toolkit [20]. Although the paradigm shift from provider-centric care to patient-practitioner partnerships is clearly emerging in practice and healthcare processes, a redefinition of patient participation is not consistently described [21]. However, students did highlight the importance of listening to patients in a non-dismissive manner, as well as valuing and respecting their experiences.

Patient safety indicators typically measured by government bodies and healthcare organizations include medication errors, falls, infection rates and adverse incidents [22–24], yet students considered broader safety issues, such as those related to transitions of care and stigma. Although ineffective transitions of care and handoffs that lead to error in information sharing have been described [25], student recognition of more nuanced safety issues, and how they will personally respond to them, is important. Stigma related to disease, ability to communicate, mental health or socioeconomic status may affect communication of safety concerns among health professionals [26] and could affect access to safe care. As such, student awareness of these broader safety issues is critical to future intervention.

Students recognized the value of health professionals advocating for patients as well as enabling them to advocate for themselves to promote optimal care. Indeed, advocacy is identified as a core competency for a number of healthcare professions (e.g. Frank et al. and the National Physiotherapy Advisory Group) [27, 28]; however, the advocacy discourse is shifting towards considering the role of partnership *with* the patient, rather than just *for* the patient [27]. Through a growing personal relationship with their health mentors and engagement in their healthcare experiences, students recognized their own potential advocacy roles and expressed a desire to be attentive to their patients, to ensure they have adequate information and to enable decision-making.

Students considered ideal practitioner characteristics through reflection on the described impact on their health mentors. Experiences ranged from errors in intervention to expressions of discomfort sharing their concerns or choices with health providers. Although students are aware of occurrence of medical errors early in their practical experiences [29], they do not often hear about the direct impact of a practitioner’s behaviour on the patient. These fresh insights created through engagement with the lived experiences could serve to guide and inform future student practice.

Collaborative patient care is central to the practice models espoused in hospitals and community care organizations and consequently, efforts to promote interprofessional education have been embraced by health profession programmes. While students recognize the value of these approaches, they do not necessarily have a full appreciation of the experiences of the patient and family members. In this teaching activity, students observed the connection between patient safety and team communication and collaboration, a link promoted by the World Health Organization [30] and the topic of numerous investigations on clinical units. Although there is agreement that collaboration is critical to quality and safe patient care, practice does not consistently follow these ideals. Opportunities for student reflection prompted by connection to patient experiences and corresponding emotive responses may impact transformative learning.

### Implications

Most health profession accreditation bodies and educational programmes mandate teaching to promote patient safety, yet traditional pedagogic approaches may not have enduring effects or be translated to future practice. Exposure to patients’ lived experiences should be investigated further to explore if and how students translate values and behaviours to future practice. Further exploration of differences in student experiences with alternate interview questions, facilitator prompts or reflective questions may be beneficial.

### Study limitations

This study was conducted in a single university environment, utilizing a convenience sample of first-year learners participating in the Health Mentor Programme. Thus, resulting themes should be interpreted within this context. A gender bias may also have affected results, with only six males in the sample of the 91 students. Focused interviews would have yielded a deeper exploration of themes presented. Additionally, the dataset may have been influenced by the discussion and reflective writing questions, as well as the format.

## Conclusions

Patient safety curricula are critical in health professions education. To date, pedagogic approaches have not capitalized on inclusion of health system users. This study demonstrated an enhanced student appreciation of issues pertaining to patient safety. Of particular interest is student appreciation of patient-practitioner partnerships, consideration of safety issues, advocacy related to enhancing safety, informing of future practice, and the value of interprofessional communication and collaboration. Further investigation of the enduring value of exposure to and lessons learned from patients may inform and supplement future health profession education.
